# White matter injury and outcomes: what is the final verdict?

**DOI:** 10.1038/s41390-025-04250-3

**Published:** 2025-07-29

**Authors:** Michelle Machie, Lina Chalak

**Affiliations:** 1 Division of Pediatric Neurology, Department of Pediatrics, University of Texas Southwestern Medical Center, Dallas, TX USA; 2 Division of Neonatal-Perinatal Medicine, Department of Pediatrics, University of Texas Southwestern Medical Center, Dallas, TX USA

Christensen et al. reports no associations between isolated T2 hyperintensities on early MR Brain and neurodevelopmental outcomes out to 3 years of age in infants with neonatal encephalopathy (NE). The study assessed 102 infants with MR Brain completed at a median age of 4 days, and quantified brain injury on early MRI using objective scoring measures: The Barkovich score was applied to rule out restricted diffusion on MRI and the Kidokoro score was applied to assess isolated T2 hyperintensities on the same MR studies.

The study by Christensen et al. derives strength from its prospective study design and the use of validated scoring mechanisms for the evaluation of neonatal brain injury including the Barkovich and Kidokoro scores. Another notable strength is the effort to minimize confounding effects in the evaluation of isolated T2 hyperintensity by excluding those infants with extensive diffusion restriction.We start with a quick overview of the objective MRI scoring tools:

1. The Barkovich score was first created to evaluate the typical basal ganglia and watershed patterns of injury on MRI in infants with perinatal asphyxia and is validated as a good predictor of longterm neuromotor and cognitive outcomes. The severity of watershed injury is graded on a scale from 0 to 5: Score 0- normal; Score 1- single focal infarction; Score 2- abnormal signal in the anterior or posterior watershed white matter; Score 3- abnormal signal in the anterior or posterior watershed cortex and white matter; Score 4- abnormal signal in both anterior and posterior watershed zones; Score 5- extensive cortical involvement.
2. The Kidokoro T2 hyperintensity score was created as a quantitative measure for diffuse excessive high signal intensity (DEHSI) in a cohort of premature infants on MR Brain obtained at term equivalent age, with follow up neurodevelopmental data out to thirteen years of age.^1^ The score stratifies severity over a 0 to 4 grade scale: Grade 0- no DEHSI throughout the white matter; Grade 1- DEHSI only within the periventricular crossroads; Grade 2- DEHSI visible in one white matter region additional to the crossroads; Grade 3- DEHSI visible in two additional regions; Grade 4- three additional regions (extensive white matter involvement).

Historically DEHSI on T2-weighted MR imaging in preterm neonates was first reported by Maalouf et al. in 2001.^2^ Since then, many of the studies examining the significance of DEHSI were in preterm infants, with the understanding that the white matter of the preterm brain is subject to selective vulnerability and high risk for injury.^3^ Over time the definition of DEHSI has broadened to involve any visually apparent high T2 signal intensity in any area of the white matter, but the diagnosis remains subjective and subject to inter-rater variability.^4,5^ The significance of DEHSI to long term neurodevelopmental outcomes in premature infants has been debated with many studies finding no significant correlation,^6–10^ but with other studies suggesting association with neuropathology and neurodevelopmental outcomes.^11,12^ The significance of DEHSI in term infants with neonatal encephalopathy remains poorly understood.^13,14^

Limitations acknowledged by the authors include lack of standardization of magnet strength in the MRI scanners and the need for neurodevelopmental follow up out to school age. Another important limitation is that the Kidokoro score was adapted from its intended use as it was validated and designed to assees preterm brain injury at term equivalent age.

Alternate MRI Scores developed specifically for the evaluation of infants with NE or hypoxic ischemic encephalopathy (HIE) include the National Institute of Child Health and Human Development Neonatal Research Network (NICHD NRN) score^15^ and the Weeke score.^16^ Both scores are validated with long term outcomes and allow for the evaluation of signal abnormalities in the basal ganglia, thalamus, and white matter.^17^ In addition, the Trivedi score includes evaluation of diffusion weighted imaging but is weighted more specifically for deep nuclear gray matter and posterior limb of the internal capsule (PLIC) injuries.^18^ These scores are all intended to be global injury severity scores and in comparison, the Kidokoro T2 hyperintensity/DEHSI score may allow for more discrete stratification of injury severity specific to this injury pattern. Application of the Kidokoro T2 hyperintensity score are needed to validate the tool in future cohorts of HIE. Additionally, false negative study findings are possible in this small Canadian cohort since no power analysis is presented, and the applicability to other cohorts with different socioeconomic and ethnicities remains unknown. We have reported in a large county hospital predominantly underserved Hispanic minorities that even a low NICHD MRI score, showing evidence of focal white matter injury (1a) was associated with cognitive deficits at 24 months of age in about 70% of patients.^19^

This study is important as early MRI after NE (DOL 3-5) is becoming more commonplace, and understanding the significance (or insignificance) of isolated T2 hyperintensities on early MRI will improve the sensitivity of our counseling and prognostic efforts for families. We like to point an important limitation is the exclusion of infants with mild encephalopathy subject of current trials (COOL Prime)^20^ with controversies in timing of the insults. Notably, antepartum asphyxia may result in T2 hyperintensity and diffusion restriction on early MRI with evolution to cystic encephalomalacia over time, and may be associated with more significant neurodevelopmental impairment^21^. Understanding the timing of asphyxia and pattern of injury on early MRI are important for accurate prognosis of long-term outcomes. Additional research is also needed to understand the significance of injury to the mamillary bodies in NE as some studies demonstrate correlation to long term cognitive outcomes.^19,20^
